# Using country of origin to inform targeted tuberculosis screening in asylum seekers: a modelling study of screening data in a German federal state, 2002–2015

**DOI:** 10.1186/s12879-019-3902-x

**Published:** 2019-04-03

**Authors:** Kayvan Bozorgmehr, Stella Preussler, Ulrich Wagner, Brigitte Joggerst, Joachim Szecsenyi, Oliver Razum, Christian Stock

**Affiliations:** 1 Department of General Practice and Health Services Research, University Hospital Heidelberg, INF 130.3, 69120 Heidelberg, Germany; 2 Department of Population Medicine and Health Services Research, School of Public Health, Bielefeld University, Bielefeld, Germany; 3 Institute of Medical Biometry and Informatics (IMBI), University Hospital Heidelberg, Heidelberg, Germany; 4 Public health authority, Section for Disease Control, Landkreis Karlsruhe, Karlsruhe, Germany; 5 Public health authority, Enzkreis, Pforzheim, Germany; 6 Department of Epidemiology and International Public Health, School of Public Health, Bielefeld University, Bielefeld, Germany; 7 Division of Clinical Epidemiology and Aging Research, German Cancer Research Center, DKFZ, Heidelberg, Germany

**Keywords:** Tuberculosis, Infection control, Public health, Migration, Asylum seekers, Screening, Efficiency, Global health, Modelling, Epidemiology

## Abstract

**Background:**

Screening programmes for tuberculosis (TB) among immigrants rarely consider the heterogeneity of risk related to migrants’ country of origin. We assess the performance of a large screening programme in asylum seekers by analysing (i) the difference in yield and numbers needed to screen (NNS) by country and WHO-reported TB burden, (ii) the possible impact of screening thresholds on sensitivity, and (iii) the value of WHO-estimated TB burden to improve the prediction accuracy of screening yield.

**Methods:**

We combined individual data of 119,037 asylum seekers screened for TB in Germany (2002–2015) with TB estimates of the World Health Organization (WHO) (1990–2014) for their 81 countries of origin. Adjusted rate ratios (aRR) and 95% credible intervals (CrI) of the observed yield of screening were calculated in Bayesian Poisson regression models by categories of WHO-estimated TB incidence. We assessed changes in sensitivity depending on screening thresholds, used WHO TB estimates as prior information to predict TB in asylum seekers, and modelled country-specific probabilities of numbers needed to screen (NNS) conditional on different screening thresholds.

**Results:**

The overall yield was 82 per 100,000 and the annual yield ranged from 44.1 to 279.7 per 100,000. Country-specific yields ranged from 10 (95%- CrI: 1–47) to 683 (95%-CrI: 306–1336) per 100,000 in Iraqi and Somali asylum seekers, respectively. The observed yield was higher in asylum seekers from countries with a WHO-estimated TB incidence > 50 relative to those from countries ≤50 per 100,000 (aRR: 4.17, 95%-CrI: 2.86–6.59). Introducing a threshold in the range of a WHO-estimated TB incidence of 50 and 100 per 100,000 resulted in the lowest “loss” in sensitivity. WHO’s TB prevalence estimates improved prediction accuracy for eight of the 11 countries, and allowed modelling country-specific probabilities of NNS.

**Conclusions:**

WHO’s TB data can inform the estimation of screening yield and thus be used to improve screening efficiency in asylum seekers. This may help to develop more targeted screening strategies by reducing uncertainty in estimates of expected country-specific yield, and identify thresholds with lowest loss in sensitivity. Further modelling studies are needed which combine clinical, diagnostic and country-specific parameters.

**Electronic supplementary material:**

The online version of this article (10.1186/s12879-019-3902-x) contains supplementary material, which is available to authorized users.

## Background

Systematic screening for tuberculosis (TB) is the “[…] systematic identification of people with suspected active TB in a predetermined target group by the application of tests, examinations or other procedures […]” [1]. Independently of the accuracy of the applied diagnostic test, the prevalence of active TB in the screened population is a key determinant of yield, numbers needed to screen (NNS) to identify one case of TB and predictive values of screening tests [2].

Asylum seekers and refugees are considered at high risk for TB infection and disease [3], mainly due to pre- and peri-migration factors favouring the transmission or re-activation of TB [4, 5]. Therefore, the majority of European countries have implemented systematic screening programmes for this group of migrants [6–8]. Screening practices among asylum seekers and refugees in Europe mostly comprise chest radiography (CXR) alone or combined with other diagnostics [9]. The overall yield of screening for active TB in asylum seekers upon-entry has been estimated to be about 0.28% in a meta-analysis of international studies [3] and about 0.35% in a meta-analysis of studies from Germany [10]. Although TB screening in asylum seekers is recommended [5, 11], there are concerns about effectiveness [12, 13] and costs of TB screening programmes [6, 14]. Asylum seekers are a risk group with high heterogeneity with respect to their individual constituencies and countries of origin [15, 16]. Screening in asylum seekers is usually not targeted, i.e. performed in a stratified manner according to risk-groups *within* this population. Evidence-based screening strategies which take the heterogeneous distribution of risk into account could help finding the same or sufficiently high number of cases using fewer resources and thus improve screening efficiency. This would be desirable especially in times of high immigration to countries with a low incidence of TB [15].

A pre-migration factor shown to affect the yield of screening in migrant populations, and thus screening efficiency, is the burden of TB in the country of origin [17]. However, although studies report country-stratified yield of screening in general migrant groups [17, 18], only few empirical studies have reported the yield of screening in asylum seekers stratified by country of origin [3, 10, 16]. The presumed link between asylum seekers’ country of origin and performance of screening programmes is suggested by clinical experience and descriptive epidemiological reports comparing the TB prevalence in the country of origin with the observed screening yield in asylum seekers. Examples are studies conducted in the Netherlands [19] and Germany [10] which compared yield of screening with TB prevalence in the country of origin reported by the World Health Organization (WHO). More analytical approaches to guide the choice of TB screening strategies have yet exclusively focused on clinical and diagnostic parameters [2], or have considered the role of country of origin but have been conducted among general migrant groups in the context of latent TB [18]. As a consequence, evidence-based guidance to inform the development of targeted screening in the heterogeneous groups of asylum seekers with respect to their country of origin is lacking. Specifically, the predictive value of asylum seekers’ country of origin for the yield of screening remains uncertain as the association between country of origin and expected yield of screening is not fully established.

We here present a modelling study to inform the development of targeted screening strategies in asylum seekers using individual and population-based parameters related to asylum seekers’ country of origin. We hypothesise that country of origin linked with information on TB burden in asylum seekers’ country of origin can be used to improve screening efficiency. Using screening data of a large German federal state, we aimed to assess the performance of the screening programme over a longer time-period with changing migration intensity. We analyse (i) the difference in yield and NNS depending on country of origin and WHO-estimated TB incidence in asylum seekers’ country of origin, (ii) the possible impact on sensitivity of screening when introducing a screening threshold, and (iii) the value of WHO-estimated TB prevalence to improve prediction accuracy of yield and therefore screening efficiency in asylum seekers.

## Methods

### Source of screening data

We used data from mandatory upon-entry screening for TB in asylum seekers between 2002 and 2015 from the main reception centre of the third largest federal state in Germany with about 10 million inhabitants (Baden Württemberg). The reception centre quasi-randomly received about 13% of newly arriving asylum seekers to Germany based on administrative quota. All newly arriving asylum seekers are registered with a unique identification number and are required to undergo a mandatory health examination according to national law [20] (§62 of the Asylum-Law in combination with §36 of the Infection Protection Act).

### Screening protocol

The health examination consists of symptom-based screening for all asylum seekers to identify communicable diseases and a *compulsory* chest X-ray (CXR) examination for all asylum seekers above 15 years (except pregnant women) regardless of the results of symptom-based screening. The protocol for children below 16 years consisted of a tuberculin skin test (2002–2010) and symptom-based screening (2011–2015). Pregnant women underwent screening for TB infection either by a tuberculin skin test or interferon-gamma release assay (IGRA) (2002–2015). For non-pregnant adults, the screening is not sequential and CXR can be regarded as the first screening test. Asylum seekers with presumptive TB undergo further diagnosis by means of a facultative chest computer scan, a sputum test (microscopy or PCR) and culture tests. Ultimately, an active TB case was defined by the fact that the physician decided to start treatment (including clinically diagnosed cases, smear-positive cases and/or culturally confirmed TB). Asylum seekers must reside in the reception centre until the final result of the mandatory TB screening is obtained and they are not transferred to counties and communes unless active TB is ruled out. Those diagnosed with active TB are put on treatment in a hospital setting and not transferred unless risk of transmission has been ruled out. Further details of the screening protocol (e.g. for children < 16 years and pregnant women) are provided elsewhere [15].

### Data source for TB in asylum seekers’ country of origin

Estimates for TB incidence and prevalence (per 100,000 population) in asylum seekers’ countries of origin were taken from the WHO Global Burden of TB database [21]. Following classifications used by the European Centre for Disease Prevention and Control [22] and de Vries et al. [19, 23], we classified asylum seekers’ countries of origin as low-, intermediate-, or high-incidence country when its WHO estimated period-averaged (1990–2014) TB incidence (Additional file 1) ranged between 0 and 20, between 21 and 50, or > 50 per 100,000, respectively. This classification was used to calculate the proportion of asylum seekers in the screening data stemming from countries with a low or intermediate incidence of TB among the total population of asylum seekers screened in each year. Further categories with higher cut-offs coded as binary variables (≥ 100, ≥ 150, ≥ 200, ≥ 250 and ≥ 300 per 100,000, respectively) were additionally used for both descriptive and analytical purposes. We further used data on TB prevalence (all forms, per 100,000 population) from WHO as external (“prior”) information for a set of 11 countries for which it was possible to calculate the country-specific observed yield from the screening data with reasonable precision (see below for details).

### Statistical analysis

We assessed the period-, country-specific and the annual yield of TB screening as well as the NNS to identify one case of TB in the German screening data as defined in Table 1.

**Table 1:** Outcomes measures, analytical perspective and statistical methods

**Yield of TB screening:** The fraction of active TB cases detected among the number of asylum seekers actively screened for TB, expressed as cases per 100,000 persons.	
**Number needed to screen (NNS)**: The number of asylum seekers that need to undergo screening in order to diagnose one person with active TB [13], calculated as the inverse of yield.	
**Bayesian perspective:** Bayesian estimation and inference generally differ from frequentist methods that are still mostly seen in clinical and public health research in treating parameters as random variables (as opposed to constants in frequentist methods) [24]. The learning process in Bayesian methods works by modifying initial probability statements about parameters (prior distributions) before observing the data to updated or posterior knowledge that combines both previous knowledge and the data at hand. It allows hypotheses to be assessed by using a collection of parameter samples from their posterior distribution. A main advantage of Bayesian methods is the probabilistic (more common sense) interpretation of the confidence interval, here termed credible interval (CrI) on parameters. Key ingredients of a Bayesian statistical model are the likelihood function, reflecting information about the parameters contained in the data, and the prior distribution, quantifying what is known about the parameters before observing data. The prior distribution and likelihood can be combined to the posterior distribution, which represents total knowledge about the parameters after the data has been observed in the following sense [38]: posterior ∞ prior × likelihood, where ∝ means “is proportional to”. When considering the occurrence of a TB case in the screening process as “success” in *n* independent trials, the prevalence may be modeled to be binomially distributed. We exploit here that the conjugate prior for the binomial distribution is the beta distribution (Additional file 1).	
**Determination of prior distributions using WHO data:** WHO reports indirect estimates with a 95% CrI, except for Gambia and Pakistan where estimates are based on population-based surveys. This information was used to derive a *Beta(p;q)* prior distribution for the TB prevalence (Additional file 1: Figure S2). According to the method of moments [39] the shape parameters p and q of the beta distribution Beta(*p;q*) were estimated on the basis of the WHO data as follows: $\overset{\wedge }{p}\kern0.5em =\kern0.5em \left(\frac{1\kern0.5em -\kern0.5em \overset{\_}{p}}{\overset{\_\kern0.5em }{\sigma^2}}\kern0.5em -\kern0.5em \frac{1}{\overset{\_}{p}}\right)\kern0.5em \overset{\_}{p^2}$ and $\hat{q}\kern0.5em =\kern0.5em \hat{p}\kern0.5em \left(\frac{1}{\overset{\_}{p}}\kern0.5em -\kern0.5em 1\right)$, where $\overline{p}$ is the mean prevalence (averaged over the years 1990 to 2014) for each country and $\overline{\sigma}$ is the mean standard deviation. The mean standard deviation $\overline{\sigma}$ is computed on the basis of the lower and upper bounds of the 95% credible intervals given by the WHO data for each country and year. It is assumed that: width (95 % credible interval) ≈ 2 × 1.96 *σ*, and therefore $\frac{\mathrm{mean}\ \left(\mathrm{width}\kern0.5em \left(95\%\mathrm{credible}\ \mathrm{interval}\right)\right)}{2\times 1.96}\kern0.5em \approx \kern0.5em \overset{\_}{\sigma }$. This gives a Beta($\widehat{p}$; $\widehat{q}$) distribution for each country.	
**Modelling country-specific probabilities of the NNS to lie above a given cut-off value** The probability that the NNS for a certain country is above a given cut-off value *t* can be calculated from the derived posterior distributions of the prevalence in each country as follows: First, we use the inverse of the expected prevalence as NNS, and then we calculate the cumulative distribution function of the prevalence at 1/t. $$ {\displaystyle \begin{array}{l}P\kern0.5em \left[ NNS\kern0.5em \ge \kern0.5em t\right]\kern0.5em =\kern0.5em P\kern0.5em \left[\frac{1}{\mathrm{expected}\kern0.5em \mathrm{prevalence}}\kern0.5em =\kern0.5em NNS\kern0.5em \ge t\right]\\ {}\kern5.12em =\kern0.5em P\kern0.5em \left[\mathrm{expected}\kern0.5em \mathrm{prevalence}\kern0.5em \le \kern0.5em \frac{1}{t}\right]\\ {}\kern5.12em =\kern0.5em F\kern0.5em \left(\frac{1}{t}\right)\end{array}} $$	

Throughout, we adopted a Bayesian perspective, thus treating parameters as random variables and considering prior information (see also Table 1 for further details) [24]. To investigate the performance of the screening programme and the suggested link between TB burden in the country of origin and the yield of screening in asylum seekers, we applied four main strategies:

1. We assessed the change over time in number of TB cases, yield per 100,000 asylum seekers, proportion of asylum seekers from low- and intermediate-incidence TB countries, and number of asylum seekers screened. We further plotted country-specific yield of screening against the number of asylum seekers screened in each category of country, stratified by the above classification based on WHO data in low−/intermediate- vs. high-incidence TB country.
2. We used Bayesian Poisson Regression with age, sex, year of screening and WHO-estimated incidence of TB (dummy: high vs. low and intermediate TB incidence) as individual-level predictors to analyse the age- and sex-adjusted difference in the yield of screening (2002–2015) between asylum seekers from countries with a WHO-estimated low and intermediate incidence of TB and those with a high incidence of TB. Flat priors were applied for all effects and the models were fit in Stata (version 14) using the Metropolis-Hastings algorithm. Five additional models with higher cut-offs of WHO-estimated incidence of TB (≥ 100, ≥ 150, ≥ 200, ≥ 250, and ≥ 300 per 100,000) were used while adjusting for the same co-variables.
3. We used period-averaged (1990–2014) WHO estimated TB incidence with their corresponding period-averaged 95% confidence intervals (Additional file 2) to calculate the impact on sensitivity of screening by introducing a screening threshold at any given value of WHO data. To this end, the number of false negative TB cases, the number of individuals not screened, and the number of false negative TB cases per 1000 individuals not screened was calculated and plotted depending on any given threshold derived from WHO data.
4. We hypothesised that TB prevalence estimates from WHO may be valuable prior information that allows improved predictions of screening yield for each single country. This is naturally accommodated under a Bayesian perspective. We assumed an informative prior distribution for the prevalence of TB in different countries on the basis of WHO data. After descriptively comparing the yield by country of origin from the screening data with the prevalence of TB in respective countries of origin based on WHO data (Additional file 1: Figure S1,), we employed a statistical model and combined the two data sources (WHO data and screening data) for predictions of yield and NNS using the R language and environment for statistical computing [25]. Finally, we provide an empirically derived overview of country-specific probabilities of NNS to assess the impact on screening efficiency when using different thresholds for screening.

### Comparison of country-specific yield with country-level WHO estimates of TB prevalence

To compare the country-specific yield of TB in asylum seekers screened in Germany (2002–2015) with country-level WHO estimates of TB prevalence reported in the WHO global TB database (1990–2014), we chose countries based on following criteria: ≥ 5 cases identified during screening *or* ≥ 5000 asylum seekers screened, *and* availability of country-level estimates in the WHO global TB database. The determination was deemed to allow estimation of screening yield and regression model parameters with reasonable precision and resulted in a manageable set of countries for reporting of stratified analyses. Eleven countries in the screening data source fulfilled the two criteria: Afghanistan, Cameroon, Eritrea, Gambia, Georgia, Iraq, Macedonia, Pakistan, Russia, Somalia and Syria.

We then determined the prior distribution based on the WHO estimates for the TB prevalence of each country and year (Table 1) and obtained the posterior distribution for TB prevalence, interpreted as *expected* TB prevalence, based on the combined data. This expected TB prevalence was used to calculate the probability that the NNS of a certain country is above a given cut-off value *t* in order to inform the choice of thresholds for or against TB screening based on asylum seekers’ country of origin (Table 1).

### Sensitivity analysis

We performed a sensitivity analysis using the most recent TB estimates for the year 2015 of the global burden of disease (GBD) study [26] as prior information instead of WHO estimates (see Additional file 1 for details).

## Results

Data on 119,037 asylum seekers from 81 countries with a median age of 24 years were available (interquartile range: 14 years) (Table 2). A total of 98 cases of pulmonary active TB were identified in the scope of upon-entry screening in the observation period over 14 years corresponding to an overall yield of 82 (68 to 100) per 100,000 and an overall NNS of 1215 (996 to 1480). The majority of asylum seekers (62.2%) were from a total of seven countries of origin. Of the 98 active TB cases, 69.3% were detected in asylum seekers from nine countries of origin (11.1% of all countries). More than three quarters of TB cases were identified in asylum seekers from countries with a WHO-estimated high incidence of TB (Table 2).

**Table 2:** Characteristics of the study population and TB cases by age group, sex, year and selected countries of origin, *N* = 119,037

Characteristic	N	%^a^	Cases with active TB detected	%^a^
Age group^b^				
< 15 years	24,249	20.4	4	4.1
15–24 years	35,390	29.8	35	35.7
25–44 years	51,111	43.0	56	57.1
45–64 years	7583	6.4	3	3.1
> 64 years	616	0.5	0	0.0
Missing data	88	0.1	0	0.0
Sex				
Female	36,872	31.0	13	13.0
Male	82,051	68.9	85	85.0
Missing data	114	0.1	2	2.0
Year				
2002–2012	36,647	30.8	41	41.8
2013	14,407	12.1	7	7.1
2014	23,916	20.1	24	24.5
2015	44,067	37.0	26	26.5
Country^a^				
Afghanistan	7433	6.2	2	2.0
Cameroon	2506	2.1	5	5.1
Eritrea	1941	1.6	9	9.2
Gambia	6971	5.9	18	18.4
Georgia	2121	1.8	5	5.1
Iraq	9955	8.4	1	1.0
Kosovo	20,336	17.1	7	7.1
Macedonia	7005	5.9	2	2.0
Pakistan	5103	4.3	7	7.1
Russia	3026	2.5	5	5.1
Somalia	1025	0.9	7	7.1
Syria	17,172	14.4	5	5.1
Other	32,401	27.2	24	24.5
Missing data	2042	1.7	1	1.0
WHO-estimated incidence of TB in country of origin (per 100,000), *N* = 116,995^c^				
≤ 50	66,735	57.0	21	21.6
> 50	50,260	43.0	76	78.4
≥ 100	40,908	35.0	67	69.1
≥ 150	35,395	30.3	61	62.9
≥ 200	19,028	16.3	32	33.0
≥ 250	11,839	10.1	19	19.6
≥ 300	4717	4.0	4	4.1

^a^Countries with either ≥5 cases of active TB detected or more than 5000 individuals screened, all others classified as “other”. Abbreviations: TB, tuberculosis ^a^ Column percent per characteristic; ^b^ Mean age [standard deviation]: 24.7 [13.0]. ^c^ Difference to *N* = 119,037 due to missing data on country of origin for *n* = 2042 individuals

**Fig. 1:**
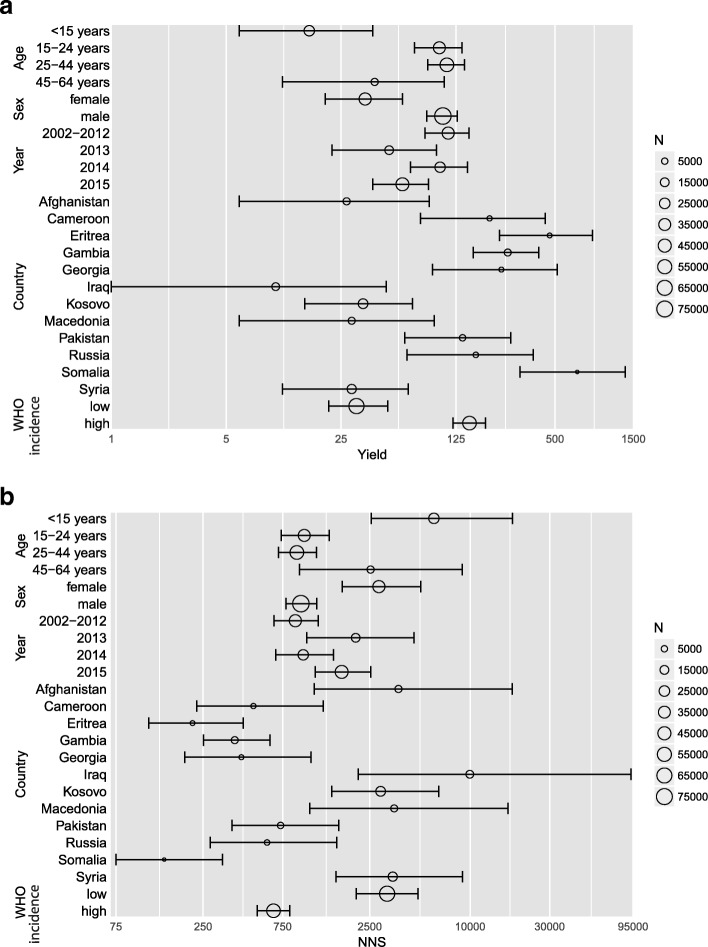
Yield and number needed to screen with credible intervals by age, sex, period, country of origin and WHO category of estimated incidence TB, *N* = 119,037 asylum seekers, 2002–2015, Germany. Legend: **a** Yield; **b** Number needed to screen, NNS. Y-Axis: log-scale. Plot size shows numbers of individuals screened

Country-specific yields were highest for asylum seekers from Somalia (lowest NNS) and lowest for asylum seekers from Iraq (highest NNS). With the exception of 2014, a lower yield of screening (and a higher NNS) was observed in 2013 and 2015 compared to the preceding time period (2002–2012), indicating a decrease in screening efficiency. Further details of yield and NNS with corresponding CrIs by age, sex, period, country of origin, and WHO category of estimated incidence of TB are presented in Fig. 1a and b (underlying data is provided in Additional file 3).

### Change in screening performance and the screened population over time

**Fig. 2:**
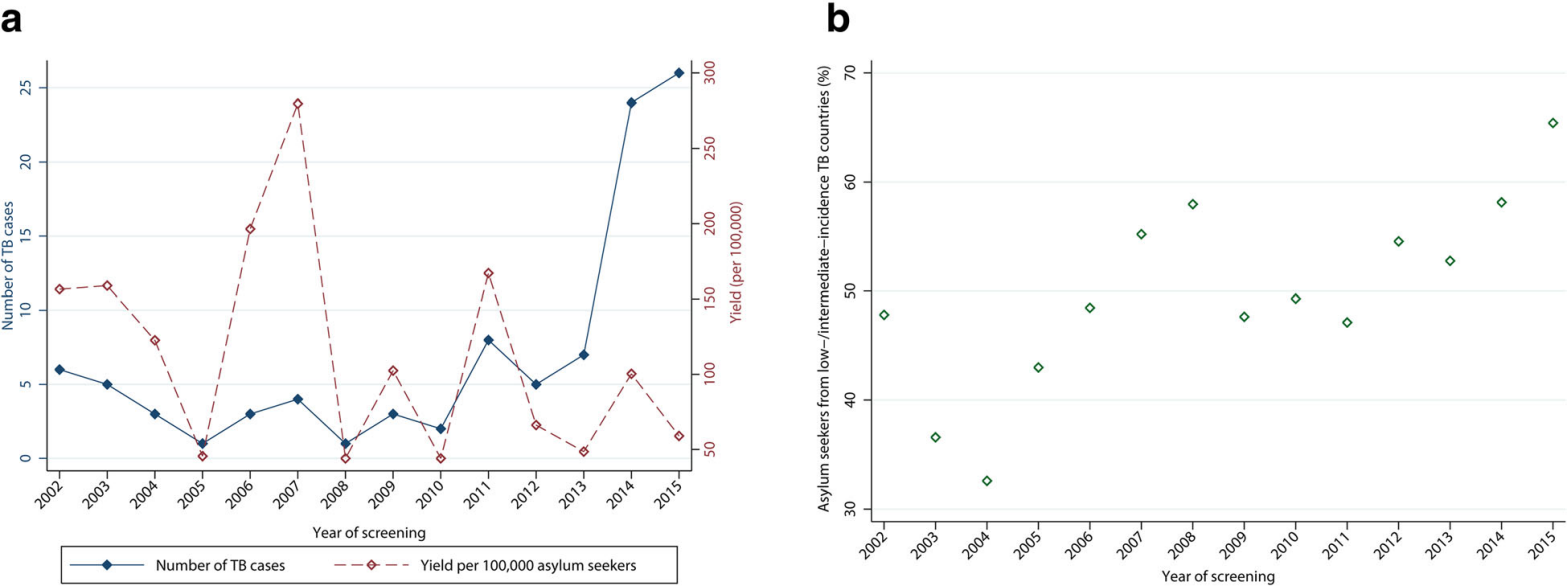
Absolute number of TB cases, yield of screening, and proportion of asylum seekers from low- and intermediate-incidence TB countries, *N* = 119,037 asylum seekers, 2002–2015, Germany. Legend: **a** Absolute number of TB cases and yield of screening by year; **b** proportion of asylum seekers from low- and intermediate-incidence TB countries by year

**Fig. 3:**
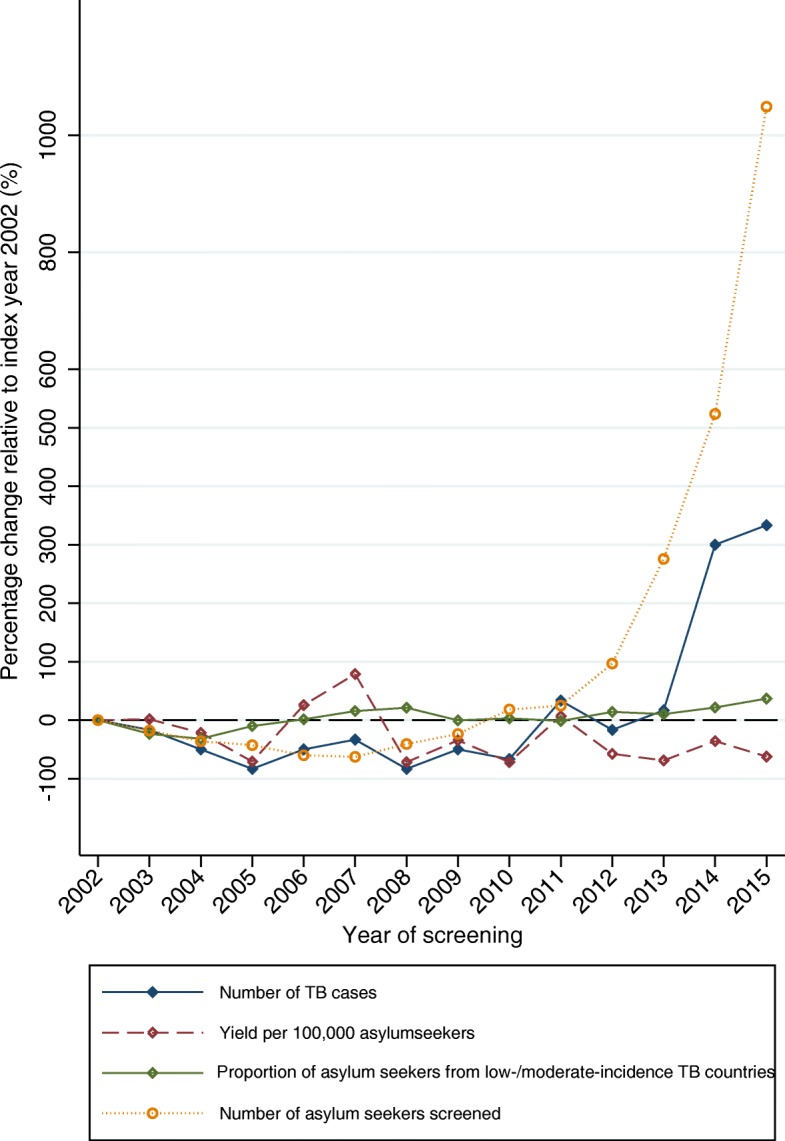
Percentage change relative to index year 2002 in number of TB cases, yield of screening, proportion of asylum seekers from low- and intermediate-incidence TB countries, and number of asylum seekers screened, *N* = 119,037 asylum seekers, 2002–2015, Germany

The annual yield varied considerably in the observation period and ranged from 44 to 279 per 100,000 asylum seekers (Fig. 2a). The proportion of asylum seekers from low- and intermediate-incidence TB countries among the screened population ranged from 32.6% in the year 2004 to 65.4% in 2015 (Fig. 2b). Compared to the index year 2002, the number of asylum seekers screened in 2015 increased more than 10-fold, TB cases (2015) by 333%, and the proportion of asylum seekers from low- and intermediate-incidence TB countries by 38.2%, while the yield (per 100,000) decreased by 62.3% (Fig. 3).

### Difference in observed yield of screening based on WHO-estimated incidence of TB

The annual proportion of asylum seekers from low- and intermediate-incidence TB countries among the screened population was negatively correlated with annual TB yield (rho: − 0.08). The overall yield of TB in asylum seekers from countries with a WHO-estimated high incidence of TB was 4.17 times the yield in those from countries with a low and intermediate incidence of TB, adjusted for age, sex and period effects (Table 3).

**Table 3:** Difference in yield of screening in asylum seekers from countries depending on the WHO-estimated incidence of TB in their country of origin, adjusted for age, sex, and period-effects, 2002–2015, Germany, *N* = 116,813

Predictor	Rate Ratio	(95% CrI)
Country of origin with a WHO-estimated high incidence of TB (> 50 per 100,000) (Reference: Country of origin with a WHO-estimated low and intermediate incidence of TB (≤ 50 per 100,000)).	**4.17**	**(2.86, 6.59)**
Age (years)	1.02	(1.00, 1.03)
Female (vs. male)	**0.44**	**(0.26, 0.72)**
Period (Reference: 2002–2012)		
2013	**0.43**	**(0.20, 0.83)**
2014	0.96	(0.62, 1,49)
2015	0.69	(0.44, 1.1)

*CrI* credible interval, *TB* tuberculosis. Difference in observations to *N* = 119,037 are due to a complete case analysis and missing data for country of origin (*n* = 2042), age (*n* = 88), sex (114). Bold figures: 95% CrI larger or smaller than 1

**Fig. 4:**
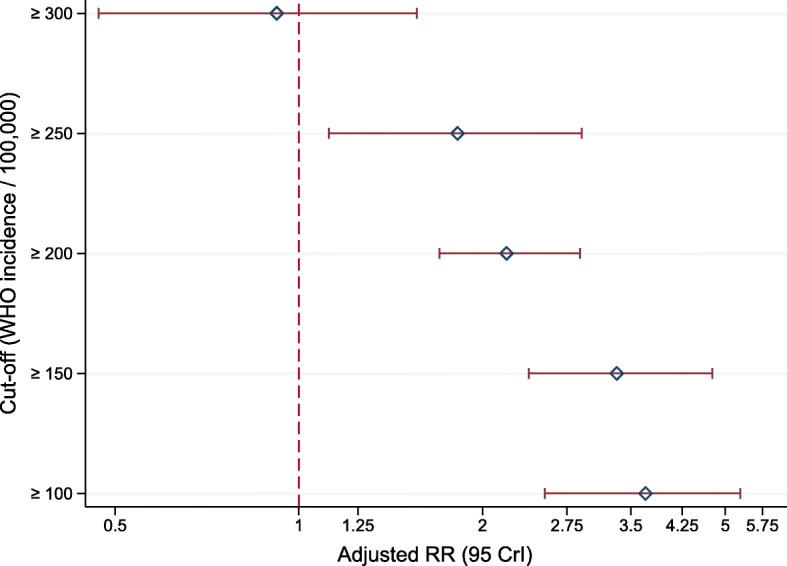
Difference in yield of screening in asylum seekers from countries depending on the WHO-estimated incidence of TB in their country of origin, adjusted for age, sex, and period-effects, 2002–2015, Germany, *N* = 116,813. Legend: Cut-off: Derived from WHO-estimated incidence of TB per 100,000. RR: Relative rate. CrI: Credible interval. Estimates derived from five distinct multiple Bayesian Poisson regression models. The reference group for each cut-off (e.g. ≥ 150) is the group of asylum seekers from countries with values of WHO-estimated TB incidence lower than the respective cut-off (e.g. < 150)

The difference in observed yield among asylum seekers from different countries of origin decreased gradually when higher cut-offs of WHO-estimated incidence of TB were used to determine comparison groups (Fig. 4).

### Comparison of country-specific observed yield with WHO estimates of TB

**Fig. 5:**
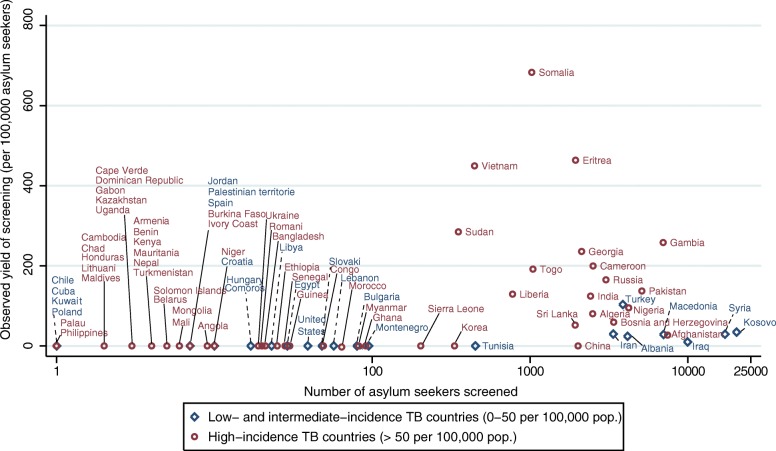
Scatter plot of observed yield of screening (per 100,000) and number of individuals screened by country of origin and WHO category of TB incidence, *N* = 116,995 asylum seekers, 2002–2015, Germany. Legend: X-axis: logarithmic scale. TB incidence in country of origin for Kosovo is taken from the review of Kurhasani et al. [40] due to missing data in WHO Global TB database

The estimated yield of screening in asylum seekers from the majority of countries was generally below 200 per 100,000, except for Cameroon, Georgia, Gambia, Sudan, Vietnam, Eritrea and Somalia. A considerable number of individuals, up to 10,000 and more asylum seekers from Iraq, Syria and the Kosovo respectively, were screened with a relatively low yield of TB (Fig. 5).

The WHO-estimated country-level prevalence of TB was close to the observed country-specific period yield of screening (2002–2015) in asylum seekers from Cameroon, Georgia, Russia, Somalia and Macedonia (Additional file 1: Figure S1). The observed period yield of screening was higher than the reported WHO estimates for asylum seekers from Eritrea and Gambia, while for asylum seekers from Afghanistan and Iraq the observed TB yield was lower (Additional file 1: Figure S1).

### Impact on sensitivity of screening depending on screening thresholds

**Fig. 6:**
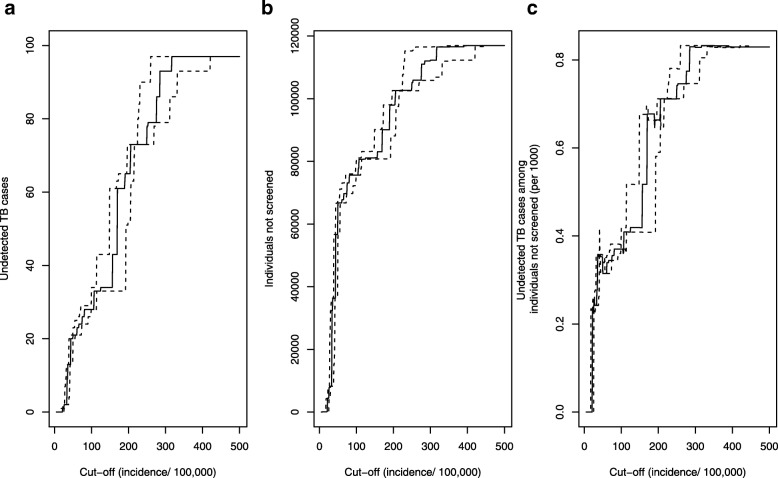
Impact of screening thresholds on sensitivity of screening, *N* = 116,995 asylum seekers, 2002–2015, Germany. Legend: **a**) Undetected cases of TB, **b**) Individuals not screened, **c**) Undetected TB cases among individuals not screened (per 1000), X-axis: Cut-off based on WHO-reported TB incidence per 100,000. Dotted lines: Cut-off calculated based on lower and upper bound of 95% confidence intervals reported by WHO. Difference to *N*=119,037 due to missing information on country of origin among =2042 asylum seekers

Introducing a threshold for screening to increase the pre-test probability would lead to “missed” TB cases (i.e. false negative individuals), and the absolute number would increase with rising screening threshold (Fig. 5a). For example, a threshold of 50 per 100,000 based on WHO-reported incidence of TB would lead to 21 (22%) undetected TB cases (Fig. 6a). However, a total of about 66,700 individuals would be exempt from screening at this threshold (Fig. 6b), corresponding to about 0.3 undetected TB cases per 1000 individuals not screened (Fig. 6c). Considering the time period (13 years), the rate of false negatives at this threshold corresponds to 2.4 undetected TB cases per 100,000 individuals per year. Increasing the threshold up-to 100 per 100,000 would lead to a marginal increase in the number of undetected TB cases per 1000 individuals, but higher thresholds come along with a higher number of undetected cases per 1000 and entail a much larger trade-off between gain in pre-test probability and loss in sensitivity (Fig. 6c).

### Comparison of prior distribution, data likelihood and posterior distribution

**Fig. 7:**
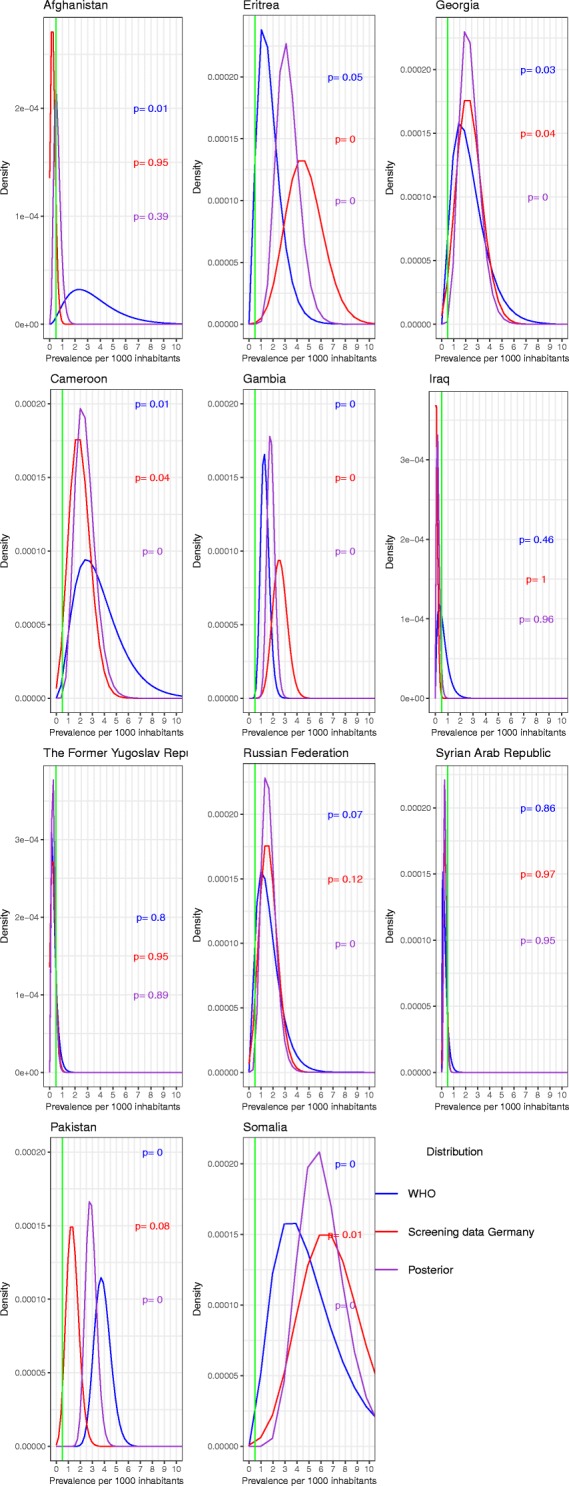
Densities of the prior distribution on the basis of the WHO data, the binomial distribution on the basis of the German screening data and the density of the posterior beta distribution of the prevalence for each country

Comparison of the different distributions of observed country-specific yield and WHO-estimated TB prevalence showed that the use of the prior information resulted in narrower density distributions of the posterior distribution and thus improved prediction for eight of the eleven countries (Fig. 7). For three countries (Afghanistan, Eritrea and Iraq) the agreement between WHO and the screening data appeared to be smaller than for the remainder of countries.

### Consequences for screening efficiency when using different NNS cut-offs for screening

**Fig. 8:**
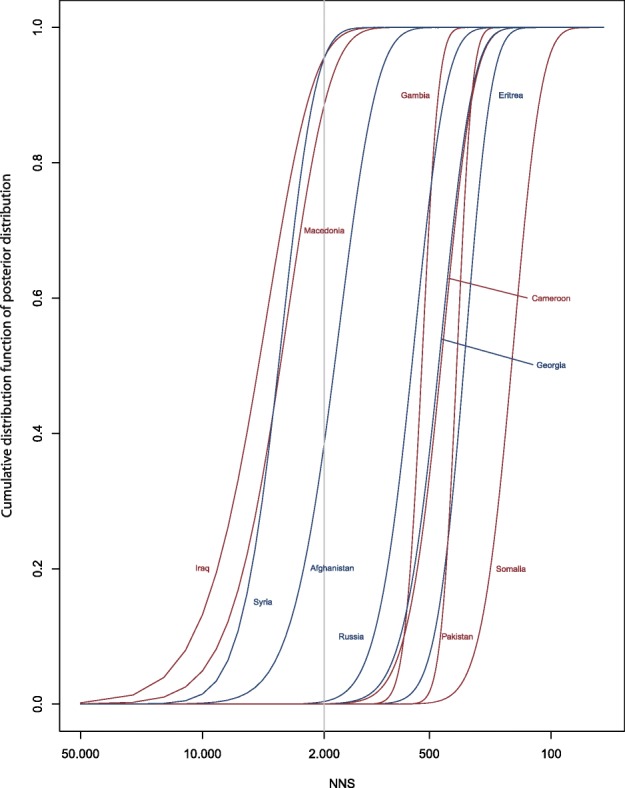
Plot of the probability that the posterior distribution of expected TB prevalence in asylum seekers from a given country of origin lies above a given NNS value

To use this information for predictions of screening efficiency, we plotted the probability to lie above a given NNS value (ranging from 0 to 50,000) for each country (Fig. 8). This showed that, for example in asylum seekers from Iraq, Macedonia and Syria, the probability is more than 80% that the NNS is higher than 2000 (a threshold that has been used in the Netherlands and Finland to decide whether or not screening should be initiated [8, 19]). The probability that the NNS is higher than 500 is less than 10% for asylum seekers from Cameroon, and close to zero for asylum seekers from Pakistan and Somalia. The expected screening efficiency in a given population can thus be estimated based on different NNS cut-offs.

### Sensitivity analysis

Using GBD estimates of TB prevalence instead of WHO data as prior improved the prediction of TB only for three countries (Eritrea, Iraq, and Pakistan). In the sensitivity analysis, the German screening data had little influence on the posterior distribution (Additional file 1: Figure S3,) and the GBD priors had substantially higher influence on the posterior distribution due to their considerably narrower uncertainty bounds (Additional file 1: Figure S4).

## Discussion

This study provides evidence that age- and sex-adjusted yield of screening for TB and corresponding NNS to identify one case of TB among asylum seekers vary strongly depending on country of origin. The dynamic changes in the population screened have implications for the performance of screening programmes, as shown here by higher NNS and lower yield after 2012 compared to preceding time periods. Being able to account for the composition and to react to possibly rapid changes in the population of asylum seekers and to corresponding TB risks is crucial for a responsive and efficient screening programme, but an adequate evidence base is required to guide decision making. We have shown that WHO-reported TB incidence is a predictor of observed yield as the age- and sex-adjusted risk of TB in asylum seekers from countries with a high incidence of TB (> 50 per 100,000) is about four times of those from countries with a low and intermediate incidence (≤ 50 per 100,000). These findings are consistent with other studies on TB in migrant populations [27, 28], although direct comparability may be limited due to differences in migrant populations, screening protocols, timing of screening, and differences in (or lack of) adjustment strategies in other studies. The discriminatory power of country of origin to determine groups of asylum seekers with higher observed yield, however, decreased with rising cut-offs above 100 per 100,000 (based on WHO-estimated incidence of TB). This is consistent with our data showing how introducing a threshold for screening to increase the pre-test probability affects the sensitivity of screening programmes. This study thus provides a useful first evidence base for decision-making with respect to the development of targeted programs.

Asylum seekers’ country of origin can inform TB screening strategies in mainly two ways. First, country-specific TB data can be used to meaningfully categorise the population to be screened according to the TB incidence in their country of origin. This information may help public health practitioners and health planners to anticipate the consequences of changes in the composition of the screened population on screening efficiency in order to react to underlying population dynamics among asylum seekers (and eventually other forced migrants). It may also help to prioritise asylum seekers from specific countries of origin for screening in times of high immigration in order to allocate scarce resources efficiently and avoid likely unnecessary diagnostics among groups of asylum seekers with low yield. The categories, however, are crude classifications, and reliance on WHO data alone may be misleading especially in fragile states or countries hit by armed conflicts. Reducing uncertainty in decision-making is thus desirable.

We show that, second, combining WHO TB prevalence data as prior information with information on observed yield from historical TB screening data reduced uncertainty of predictions. This approach allowed modelling the expected country-specific yield of TB (posterior distribution) in asylum seekers from 11 countries with greater precision. This information can be practically useful to derive country-specific probabilities for the NNS conditional on a given threshold to inform TB screening programs or guide cost-effectiveness analysis.

### Implications for screening programmes

Only few countries perform targeted screening based on TB incidence in the country of origin (e.g. the United Kingdom and Switzerland), and incidence thresholds at which screening is initiated vary, ranging from > 15, > 40, > 50 to > 100 per 100,000 population [4, 8]. In Germany, no targeted approach exists and all migrants in collective accommodation centers aged 16 years and above undergo compulsory screening (except for pregnant women and children who may be considered for screening based on sub-national regulations [29]). The results of our study question such indiscriminate screening policies. Other countries use NNS thresholds to decide whether or not to continue or discontinue screening in a given population. In the Netherlands, for example, screening is ceased at an estimated NNS > 2000 [19]. These thresholds are, however, to a certain degree arbitrary and based on practical experience and the question is how to best choose a threshold. Making decisions about targeted screening programmes requires that questions around sensitivity, timing of screening, and efficiency and cost-effectiveness are addressed.

Our study provides an empirically derived alternative to previous decision-making for or against screening in this context. Limiting screening to a smaller fraction of asylum seekers will, by necessity, miss some rare cases originating from low-incidence countries. The important normative question at the societal or health system level is therefore what is valued as a good or acceptable balance between sensitivity of screening and efficiency and cost-effectiveness of screening. Introducing a threshold means to abandon the traditional idea that entry screening should detect *all* TB cases among migrants, which is likely to be an inefficient and perhaps also an unrealistic target. Based on the analysis of change in sensitivity when increasing the pre-test probability, our data suggests that a threshold, based on WHO-reported TB incidence between 50 and 100 per 100,000, would entail the lowest trade-off between loss in sensitivity and efficiency of screening. When introducing a screening threshold at 50 per 100,000, the number of false negative individuals, i.e. asylum seekers with TB going unnoticed, per 1000 individuals is relatively low (although it cannot be ruled out that these would have been also identified due to clinical symptoms or contact investigations). While screening *all* individuals upon-entry is an approach that might be feasible in times of low immigration, it is technically, practically, ethically, and economically questionable, especially in times when numbers of immigrants peak. Passive case finding approaches and improved access to primary care [30] could complement a targeted approach at such a threshold to ensure that the few individuals with TB among those exempt from screening are noticed as soon as an infection turns into disease and becomes symptomatic. If the purpose of a programme is to increase efficiency, the threshold could probably be even increased to 100 per 100,000 without substantial loss in sensitivity.

Furthermore, the estimated country-specific proportion of detected TB cases conditional on NNS-based cut-offs allows assessing the impact on screening efficiency when using *different* NNS thresholds for screening of asylum seekers from a given country. The data provided by our study can be used to calculate such targets and estimate marginal costs of finding additional TB cases in asylum seekers from low-incidence countries to guide such decisions. Even with universal screening and more if screening is selective, the country has to expect that some TB cases will be undetected and will appear after entry. Research in a large cohort of immigrants to the United Kingdom has shown that the risk of TB incidence among immigrants screened negative before entry is highest during the first two to 4 years after immigration [17]. Furthermore, the proportion of cases detected before entry are relatively low compared to those developing TB after immigration [17]. Data from Germany is consistent with these findings, showing that cases detected upon entry are only a small fraction of cases becoming incident in the following years [31]. Providing universal access to health care and social protection [32] and complementing targeted upon-entry screening with adequate (i.e. culturally, ethically and economically acceptable) programmes for post-migration follow-up for TB [16, 33] is thus of crucial importance, also for TB control in Germany.

Our study adds further complexity to the question of “pre-entry, post-entry or no tuberculosis screening” [11] among immigrants by considering asylum seekers as a socially constructed, heterogeneous group [16]. Asking *whom* to screen among asylum seekers instead of *whether or not* to screen [11] all would be of special importance for countries at the external borders of the EU such as Italy, Greece and Spain with a high number of forced migrants arriving every year. These countries currently apply an ‘all or nothing principle’ with respect to TB screening (Greece and Spain actively screening all, and Italy none of the incoming refugees [8, 9]). A more nuanced approach would consider the expected TB prevalence based on asylum seekers’ countries of origin. Such an approach should be closely linked with the question of the design and optimal timing for post-migration follow-up [17, 33] screening for asylum seekers from countries at highest risk for TB [16].

Beyond efficiency, our study has also implications with respect to equity and ethical aspects. Screening strategies that take into account country-specific risk avoid overprovision of services [34], allow to allocate resources where needs are highest (vertical equity), and are therefore an essential step towards tailored and appropriate high-value care [35, 36] for asylum seekers. Targeted screening is, however, a double-edged sword. Ethical implications from strategies targeting asylum seekers based on country of origin are to avoid stigmatisation. This requires that clinicians, policy makers, politicians and public health services effectively communicate the fact that these groups area “at higher risk” of having TB, not “a higher risk” for importing TB [37].

### Future research

Prediction algorithms for targeted screening could be further improved by combining clinical, diagnostic [2, 13] and country-specific parameters. A combination with age, sex and socioeconomic status could further improve estimates of expected yield. This is especially relevant to further decrease the potential loss in sensitivity, and reduce the trade-off between sensitivity and high pre-test probability. Clinical information on co-morbidities (e.g. HIV, or diabetes) and socioeconomic factors were, however, not available in our data. The empirical derivation of such algorithms and their validation is further complicated because TB is a rare disease (thus, large samples are required) and available data often contain little detail on personal characteristics. We were able to estimate parameters for only 11 of 81 countries with reasonable precision. Pooling of large datasets across countries with comparable screening protocols is needed to enable further data analysis, especially as the overall yield in our study was lower than those reported in other studies on asylum seekers [3, 10]. This would allow replicating and applying our approach to other countries of origin. Initiatives such as the E-DETECT TB project co-funded by the European Commission [8] may be instrumental to this end. Furthermore, cost-effectiveness studies based on the data on thresholds and NNS provided in this study, or in studies with a larger sample size with more TB cases could be performed to generate further guidance for the decision-making towards targeted screening programmes.

### Strengths and limitations

We used a mono-centric data source spanning 14 years of upon-entry TB screening in one of the largest German federal states, which is comparable in size and population to Belgium or the Netherlands. The sample can be seen as representative with respect to age and sex distribution for asylum-seekers in Germany. The composition of the target population with respect to countries of origin may vary between federal states due to administrative procedures in the asylum process. However, this variation affects only few countries of origin, and the majority of asylum seekers are distributed to federal states based on a quasi-random administrative process [29]. It is hence likely that the composition of the population over 13 years resembles those of Germany as a whole, although direct comparison with administrative data provided by the Federal Agency for Migration and Refugees is difficult.

We only focused on active TB cases as the primary aim of upon-entry screening is to avoid transmissions in asylum seekers’ shelters. Including asylum seekers with latent TB in the analysis would have increased the yield estimates, but would have been less relevant for informing screening programmes.

Despite the large sample used the generalizability to other EU countries may be limited due to the heterogeneity in screening protocols [8]. It may also be the case that countries of first arrival of asylum seekers in Southern Europe have different (and higher) screening yields. However, we are not aware of any systematic analysis of differences in screening yield depending on the migration trajectory.

Given the low yield of TB screening and the uncertainty around estimates of observed screening yield, external evidence on TB burden should generally be considered in decisions for or against screening of asylum seekers from a given country since the uncertainty can be reduced. Depending on the general agreement between prevalence in country of origin and empirical screening data this may be sensible for some countries (e.g. Syria), but not for all countries (e.g. Afghanistan). It generally appears sensible where no or very sparse evidence exists from screening studies, where estimated TB prevalence and observed screening yield are not in conflict, and where no evidence exists that the screened population is substantially different from the general population with respect to TB risk. It might be sensible to add some “extra-uncertainty” to the country-specific prior (i.e., not applying the prevalence as the prior distribution 1:1 but to make it less informative) to consider the possibility that asylum and general populations differ with respect to TB risk. We have shown that WHO data is more reasonable to use as prior information instead of GBD estimates. The uncertainty in WHO data is a study limitation, and using period-averaged estimates as proxy of country-specific TB risk may not reflect the “true” risk at time of emigration. But this approach seemed as both practicable and reasonable for the vast population in our sample. Although GBD estimates appear more robust, and thus intuitively better, their narrow uncertainty bounds imply such a high certainty that no other source of information (in this case observed TB prevalence taken from screening data) was able to influence the distribution of data. This was not unproblematic as the deviance between observed screening data and prior information was higher for GBD data than for WHO data. The reason for this may be the use of period prevalences for both WHO (1994–2014) and German screening data (2002–2015), while using point prevalences (2015) for GBD data.

## Conclusion

Information on TB in the country of origin of asylum seekers can guide the choice of screening thresholds. A threshold based on WHO-reported incidence between 50 and 100 per 100,000 increases the pre-test probability (and screening efficiency) substantially while keeping the loss in sensitivity at an acceptable level. Consideration of WHO data on TB prevalence in the country of origin of asylum seekers can further improve the precision of estimates of screening yield. This helps to reduce uncertainty in making an informed decision for or against TB screening in asylum seekers from a given country, and allows to model country-specific NNS thresholds. We demonstrate how this can be achieved as a step towards a (more) targeted and more efficient TB screening in asylum seekers. Countries with indiscriminate screening programmes should consider this evidence to enhance the performance of their screening strategies.

## Additional files

Additional file 1: Technical appendix. Technical appendix containing additional details related to methods, data sources, and analysis. (PDF 542 kb)

Additional file 2: Period averaged incidence of tuberculosis (per 100,000) and 95% confidence intervals reported by WHO, 1990–2014. Overview of period averaged incidence of tuberculosis (per 100,000) and 95% confidence intervals reported by WHO (1990–2014) based on own calculations for countries used in this study. (PDF 113 kb)

Additional file 3: Yield of screening and number needed to screen. Yield of screening for active TB per 1000 screened individuals and number needed to screen to detect 1 case of TB with 95% credible intervals, stratified by age group, sex, year of screening, and asylum seekers’ country of origin. (PDF 77 kb)

## Abbreviations

aRR: Adjusted Rate Ratio
CrI: Credible interval
CXR: Chest X-ray
EEA: European Economic Area
EU: European Union
GBD: Global Burden of Disease
NNS: Number needed to screen
PCR: Polymerase chain reaction
TB: Tuberculosis
WHO: World Health Organization
