# Supplementary material for: Using country of origin to inform targeted tuberculosis screening in asylum seekers: a modelling study of screening data in a German federal state, 2002–2015
Source: BMC Infect Dis. 2019 Apr 3;19:304. doi: 10.1186/s12879-019-3902-x (PMC6448304; doi:10.1186/s12879-019-3902-x)
Supplement: Supplementary file 1 — Technical appendix. Technical appendix containing additional details related to methods, data sources, and analysis. (PDF 542 kb) [file 12879_2019_3902_MOESM1_ESM.pdf]

Supplementary File 1 - Technical Appendix

Comparison of country-specific yield with country-level WHO estimates of TB prevalence

Figure S1: Country-level WHO prevalence of TB (per 1,000 inhabitants) by country of origin of asylum seekers and period averaged observed yield of screening for selected countries of origin of asylum seekers in Germany (2002-2015)

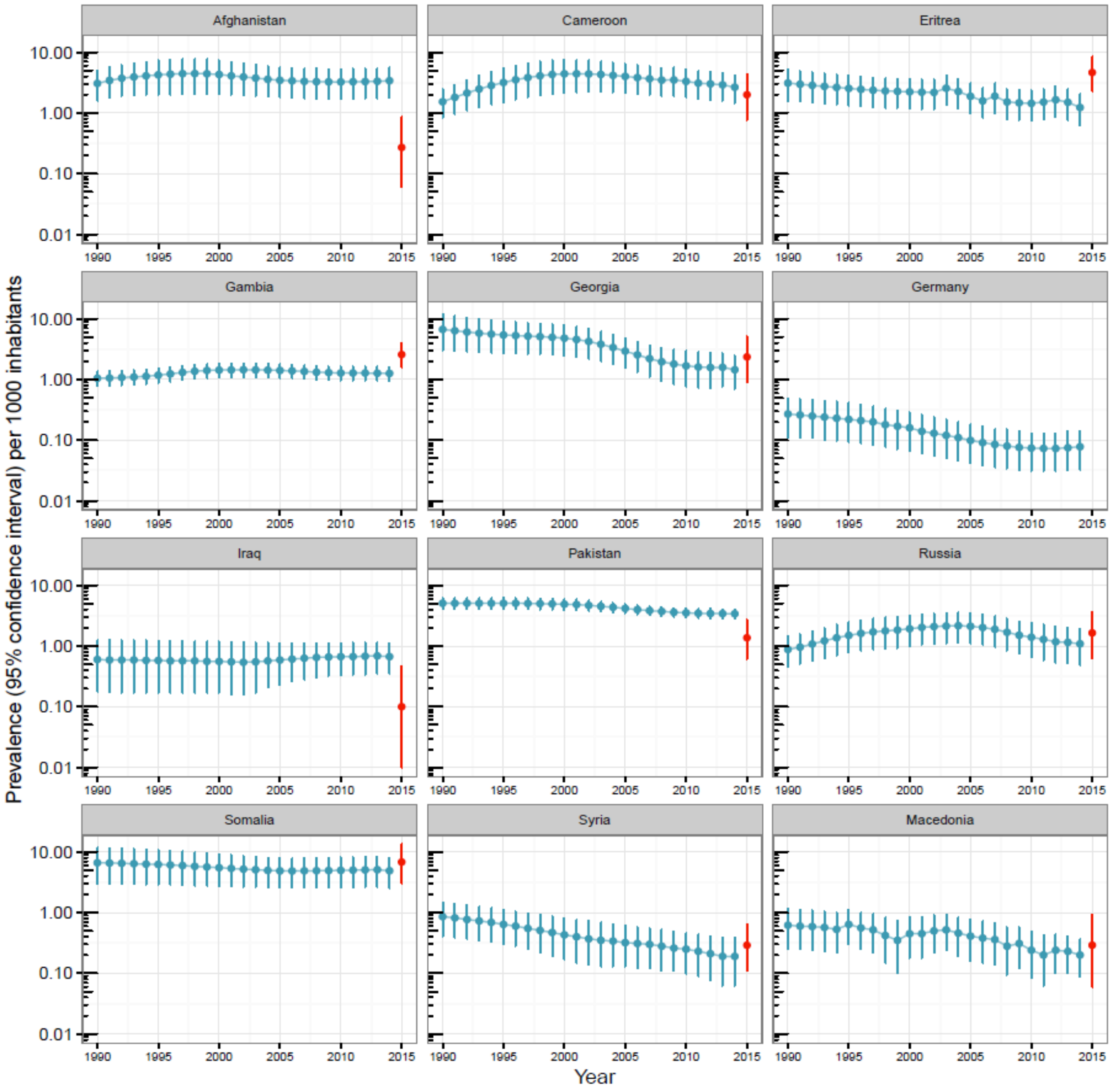

Legend: Countries with either ≥5 cases of active TB detected or more than 5000 individuals screened. The points and error bars represent point estimates of prevalence (yield) along with 95% credible intervals. Blue error bars: WHO estimates of TB prevalence (per 1,000 inhabitants). Kosovo not depicted due unavailability of corresponding data in the WHO database. Country-level TB prevalence in Germany was included here for comparison. Red error bars: Period averaged (2002-2015) country-specific observed yield of screening in German screening data.

Choosing a model

The Bayesian design of experiments includes a concept called 'influence of prior beliefs'. Therefore, the key ingredients are the likelihood function, reflecting information about the parameters contained in the data, and the prior distribution, quantifying what is known about the parameters before observing data. The prior distribution and likelihood can be combined to the posterior distribution, which represents total knowledge about the parameters after the data has been observed in the following sense [1]:

$$\text{posterior} \propto \text{prior} \times \text{likelihood},$$

where $\propto$ means "is proportional to".

When considering the occurrence of a TB case in the screening process as "success" in *n* independent trials, the prevalence may be modeled to be binomial distributed. The conjugate family of priors for a binomial observation is the beta family. When considering

$$y|\pi \sim \mathcal{B}in(n,\pi)$$

with

$$\pi \sim \mathcal{B}eta(p,q),$$

the likelihood function and prior density function are given by

$$f(y|\pi) = \binom{n}{y}\pi^{y}(1-\pi)^{n-y},$$
$$g(\pi;p,q) = \frac{\Gamma(p+q)}{\Gamma(p)\Gamma(q)}\pi^{p-1}(1-\pi)^{q-1},$$

for $0 \leq \pi \leq 1$, $y = 1,\ldots,n$ and $p, q > 0$, where Γ denotes the gamma function. The density of the posterior distribution can be calculated directly:

$$\begin{aligned} g(\pi|y) &\propto g(\pi) \times f(y|\pi) \\ &\propto \frac{\Gamma(p+q)}{\Gamma(p)\Gamma(q)}\pi^{p-1}(1-\pi)^{q-1} \times \binom{n}{y}\pi^{y}(1-\pi)^{n-y} \\ &\propto \pi^{p+y-1}(1-\pi)^{q+n-y-1}. \end{aligned}$$

This can be recognized as the shape of a beta distribution with parameters p' = p+y and q' = q+n-y.

Determination of the prior distribution on the basis of the WHO data

Choosing a prior distribution

The WHO data provides estimates for the TB prevalence of each country and year based on indirect estimates with a 95% credible interval (except for Gambia and Pakistan where estimates are based on population-based surveys). This information was used to derive a Beta(p;q) prior distribution by the method of moments. Skew distributions were convenient to be used, as the data did not seem to be symmetric distributed (compare Figure S2).

Beta prior distribution

The shape parameters p and q of the beta distribution Beta(p;q) are estimated on the basis of the WHO data accordingly to the method of moments:

$$\tilde{p} - \left(\frac{1-\bar{p}}{\bar{\sigma}^2} - \frac{1}{\bar{p}}\right)\bar{p}^2$$

$$\tilde{q} - \tilde{p}\left(\frac{1}{\bar{p}} - 1\right),$$

where $\bar{p}$ is the mean prevalence (averaged over the years) for each country and $\bar{\sigma}$ is the mean standard deviation. The mean standard deviation $\bar{\sigma}$ is computed on the basis of the lower and upper bounds of the 95% credible intervals given by the WHO data for each country and year. It is assumed that:

$$width(95\%\ credible\ interval) \approx 1.96\ \sigma$$

And therefore

$$\frac{mean(width(95\%\ credible\ interval))}{1.96} \approx \bar{\sigma}$$

This gives a Beta($\tilde{p}$; $\tilde{q}$) distribution for each country. We used density graphs to check whether the beta prior distributions match the observed estimates and intervals, and determined the equivalent sample size (i.e. the amount of information about the parameter from the prior that is equivalent to the amount from a random sample of that size) [2] .

Some precautions before using the prior should be taken into consideration. The shape should look reasonably close to the prior belief, that is the data. Therefore, graphs of the data and distributions are helpful. Figure S2 (below) shows the densities of the fitted prior distributions in comparison with the WHO data.

Figure S2: Densities of the fitted prior distributions and WHO data for TB prevalence (per 100,000) in the selected eleven countries

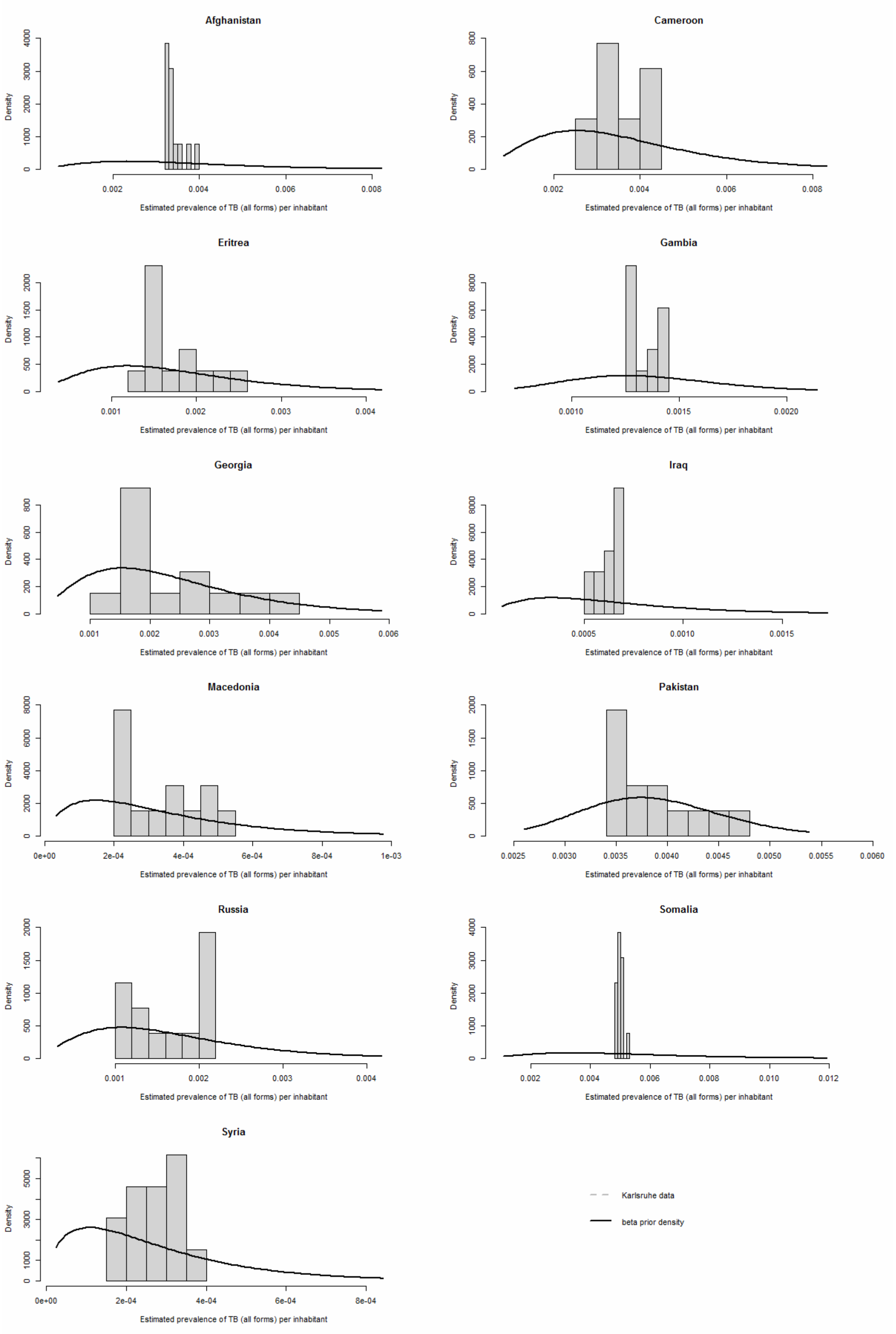

Furthermore, we note, that the sample proportion $\pi = {}^{y}/_{n}$ from a Bin(π ;n) distribution has variance equal to $\frac{\pi(1-\pi)}{n}$. To calculate the equivalent sample size, this variance will be equated at π_0 – the prior mean – to the prior variance:

$$\frac{\pi_0(1-\pi_0)}{n_{eq}} = \frac{pq}{(p+q+1)(p+q)^2}.$$

Since $\pi_0 = \frac{p}{p+q}$, the equivalent sample size is $n_{eq} = p + q + 1$. It says that the amount of information about the parameter from the prior is equivalent to the amount from a random sample of that size. It should always be checked if this is unrealistic high. So, always ask "Is the prior knowledge about p really equal to the knowledge about p that one would obtain if checking a random sample of size n_{eq}?" If not, the prior standard deviation should be increased. Otherwise, too much prior information will be put about the parameter relative to the amount of information that will come from the data[2].

Table S1: Equivalent sample size based on the beta prior distribution for WHO data and data of the Global Burden of Disease study

Country	Equivalent sample size WHO	Equivalent sample size GBD
Afghanistan	906	114,220
Cameroon	947	83,658
Eritrea	1,790	69,932
Gambia	10,757	116,794
Georgia	1,193	140,127
Iraq	3,374	182,163
Macedonia	5,395	225,165
Pakistan	8,065	92,603
Russia	1,655	224,702
Somalia	629	90,406
Syria	5,877	132,075

Sensitivity analysis

Prior information, screening data and posterior: densities in comparison

Figure S3: Densities of the prior distribution on the basis of the Global Burden of Disease estimates of TB prevalence, the binomial distribution on the basis of the German screening data and the density of the posterior beta distribution of the prevalence for each country

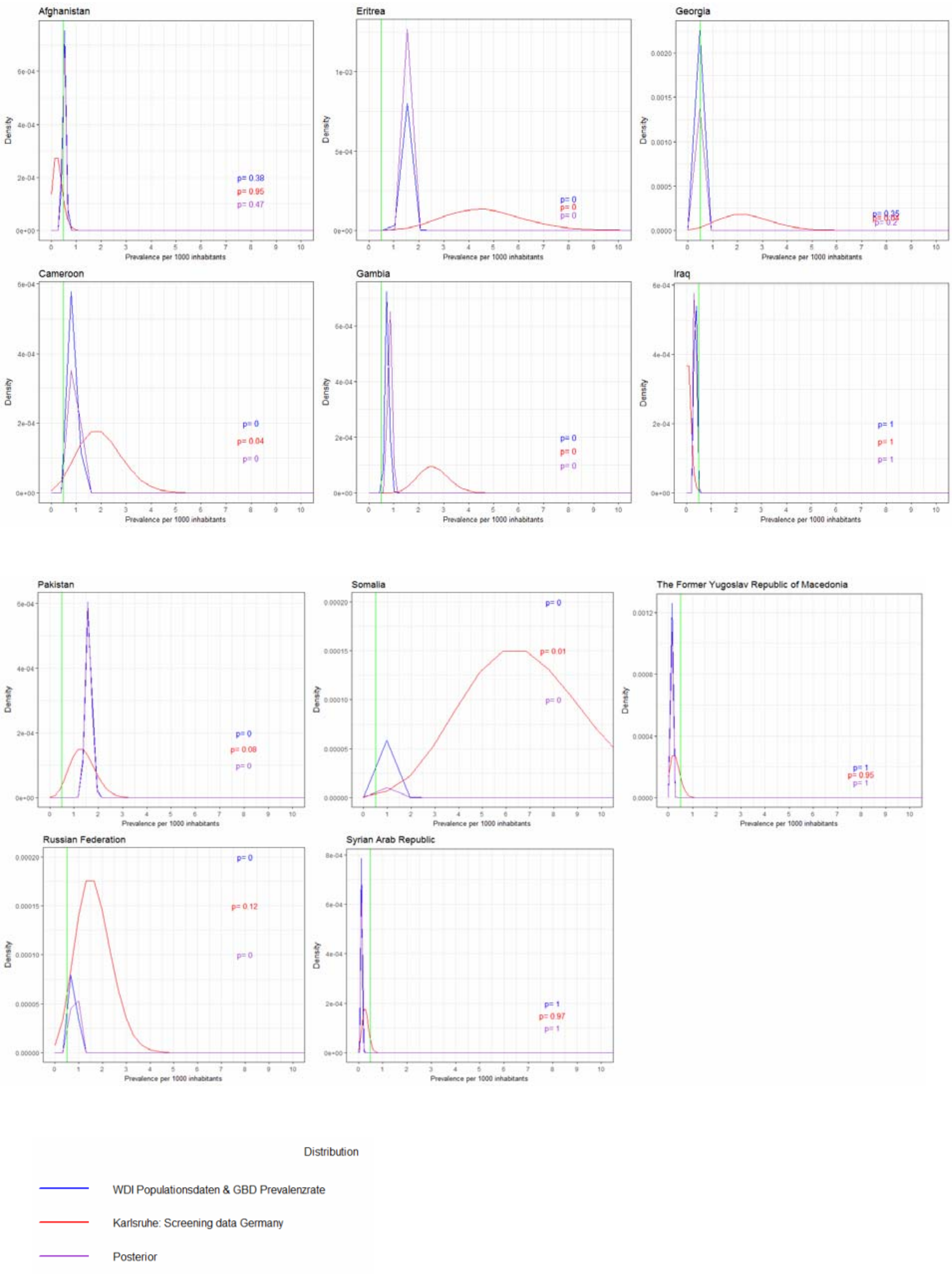

Figure S4: Comparison of estimated prevalence of TB per 100,000 and uncertainty bounds by source of data

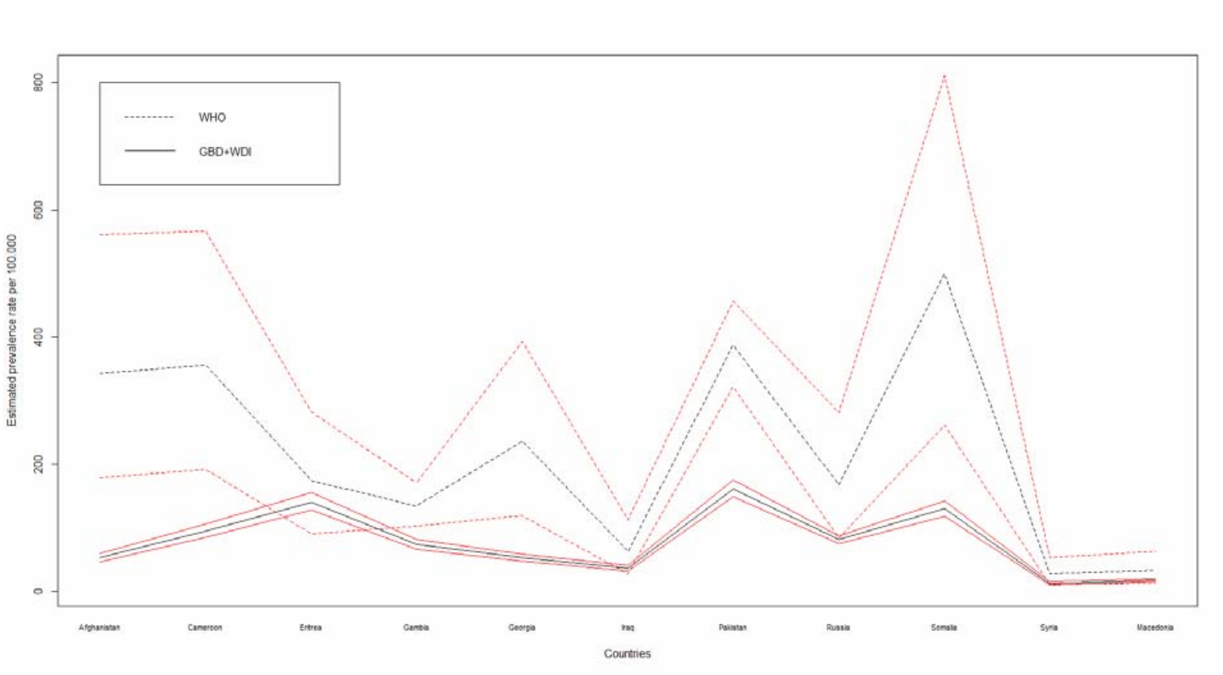

WHO: World Health Organization, GBD-WDI: TB prevalence rates calculated by Global Burden of Disease data and World Development Indicators (population data)

References

(1) Ambrosius WT. Topics in biostatistics. New Jersey: Springer; 2007.

(2) Bolstad WM, Curran JM. Introduction to Bayesian Statistics. 3 ed. Wiley; 2016.
