# Supplementary material for: Using country of origin to inform targeted tuberculosis screening in asylum seekers: a modelling study of screening data in a German federal state, 2002–2015
Source: BMC Infect Dis. 2019 Apr 3;19:304. doi: 10.1186/s12879-019-3902-x (PMC6448304; doi:10.1186/s12879-019-3902-x)
Supplement: Supplementary file 2 — Period averaged incidence of tuberculosis (per 100,000) and 95% confidence intervals reported by WHO, 1990–2014. Overview of period averaged incidence of tuberculosis (per 100,000) and 95% confidence intervals reported by WHO (1990–2014) based on own calculations for countries used in this study. (PDF 113 kb) [file 12879_2019_3902_MOESM2_ESM.pdf]

Supplementary file 3: Period averaged incidence of tuberculosis (per 100,000) and corresponding 95% confidence intervals reported by WHO, 1990-2014 (own calculations)

Country	Incidence	95 % CI (lower bound)	95 % CI (upper bound)
Afghanistan	189.12	172.84	206.28
Albania	21.2	17.72	25.28
Algeria	80.2	70.52	90.32
Angola	321.52	208.08	459.2
Armenia	53.88	50.6	57.32
Bangladesh	225	204.64	246.12
Belarus	68.24	62.64	74.28
Benin	84.88	69.04	102.36
Bosnia and Herzegovina	74.92	54.48	98.4
Bulgaria	49	46.48	51.76
Burkina Faso	69.68	65.16	74.16
Cambodia	520.72	478.08	565.24
Cameroon	249.08	230.08	268.88
Cape Verde	157.64	143.52	172.6
Chad	140.44	129.64	151.52
Chile	25.48	22.4	28.76
China	104.76	98	111.88
Comoros	39.88	33.04	47.56
Congo	334.28	305.72	364.52
Croatia	37	32.44	41.88
Cuba	13.584	12.24	15.04
Dominican Republic	96.08	87.2	105.52
Egypt	24.64	23.08	26.32
Eritrea	156.48	113.84	205.64
Ethiopia	351.12	294	413.56
Gabon	439.88	406.24	474.92
Gambia	169.68	148.72	192.24
Georgia	205.52	196.56	215.12
Ghana	202.88	94.48	352.4
Guinea	218.88	202.24	235.92
Honduras	87.04	75.04	99.76
Hungary	30.2	26.56	34.24
India	204.76	195.12	214.72
Iran	25.92	22.8	29.12
Iraq	49.16	43.16	55.64
Ivory Coast	286.56	270.6	302.76
Jordan	8.804	7.732	9.976
Kazakhstan	134.6	87.04	192.28
Kenya	259.04	252.56	265.6

Korea	390.4	335.2	450.28
Kosovo*	43.7	38.79084	49.05814
Kuwait	27.32	23.84	30.8
Lebanon	19.72	17.36	22.4
Liberia	253	230.72	276.88
Libya	40	33.12	47.76
Lithuania	81.32	77.08	85.76
Macedonia	33.72	29.56	38.12
Maldives	73.72	66.56	81.56
Mali	71.76	70.32	73.2
Mauritania	238.24	194.16	287.12
Mongolia	262	241.4	283.56
Montenegro	23.3	20.6	26.6
Morocco	119.68	111.52	127.96
Myanmar	397.48	366.32	430.16
Nepal	162.72	148.12	178.12
Niger	193.76	179.76	208.16
Nigeria	316.36	227.36	420.52
Pakistan	275.44	224.6	331.4
Palestinian territories	7.904	6.924	8.888
Philippines	358.52	327.04	391.8
Poland	33.12	29	37.6
Romania	149.96	138.76	161.4
Russia	106.16	99.24	113.36
Senegal	144.84	133.84	156.28
Sierra Leone	299.32	251.72	351.32
Slovakia	20.908	18.44	23.776
Solomon Islands	178.16	139.4	222.32
Somalia	284.96	259.44	311.76
Spain	21	18.52	23.8
Sri Lanka	65.96	60.28	72.32
Sudan	124.32	67.44	198.64
Syria	33.72	27.2	41.08
Togo	60.68	49.36	73.16
Tunisia	27.6	26	29.16
Turkey	35.8	31.4	40.52
Turkmenistan	146.08	120.12	174.64
Uganda	382.64	322.04	448.92
Ukraine	99.4	92.08	107.12
United States	6.876	6.024	7.796
Vietnam	190.28	167.6	214.68

*Data for Kosovo was taken and re-calculated from Kurhasani X, Hafizi H, Toci E, Burazeri G. Tuberculosis Incidence and Case Notification Rates in Kosovo and the Balkans in 2012: Cross-country Comparison. *Materia Socio-Medica* 2014; **26**(1): 55-8.
