# Supplementary material for: Using country of origin to inform targeted tuberculosis screening in asylum seekers: a modelling study of screening data in a German federal state, 2002–2015
Source: BMC Infect Dis. 2019 Apr 3;19:304. doi: 10.1186/s12879-019-3902-x (PMC6448304; doi:10.1186/s12879-019-3902-x)
Supplement: Supplementary file 3 — Yield of screening and number needed to screen. Yield of screening for active TB per 1000 screened individuals and number needed to screen to detect 1 case of TB with 95% credible intervals, stratified by age group, sex, year of screening, and asylum seekers’ country of origin. (PDF 77 kb) [file 12879_2019_3902_MOESM3_ESM.pdf]

Supplementary file 1 – Yield of screening and number needed to screen

Characteristic	Yield of active TB per 1000 screened (95% CrI)	Number needed to screen to detect 1 case of active TB (95% CrI)
Age group		
<15 years	0.16 (0.06, 0.39)	6,062 (2,550, 17,959)
15-24 years	0.99 (0.70, 1.36)	1,011 (736, 1,427)
25-44 years	1.10 (0.84, 1.41)	913 (708, 1,196)
45-64 years	0.40 (0.11, 1.06)	2,528 (947, 8,974)
>64 years	--	--
Sex		
Female	0.35 (0.20, 0.59)	2,836 (1,707, 5,060)
Male	1.04 (0.83, 1.27)	965 (785, 1,200)
Year		
2002-2012	1.12 (0.81, 1.50)	894 (666, 1,228)
2013	0.49 (0.22, 0.95)	2,058 (1,048, 4,601)
2014	1.00 (0.66, 1.47)	997 (681, 1,516)
2015	0.59 (0.39, 0.85)	1695 (1,175, 2,534)
Country		
Afghanistan	0.27 (0.06, 0.86)	3,716 (1,159, 17,883)
Cameroon	2.00 (0.76, 4.37)	501 (229, 1,313)
Eritrea	4.64 (2.30, 8.45)	216 (118, 435)
Gambia	2.58 (1.59, 3.99)	387 (251, 630)
Georgia	2.36 (0.90, 5.16)	424 (194, 1,111)
Iraq	0.10 (0.01, 0.47)	9,955 (2,130, 92,262)
Kosovo	0.34 (0.15, 0.68)	2,905 (1,480, 6,494)
Macedonia	0.29 (0.06, 0.92)	3,502 (1,092, 16,854)
Pakistan	1.37 (0.61, 2.69)	729 (372, 1,629)
Russia	1.65 (0.63, 3.62)	605 (276, 1,585)
Somalia	6.83 (3.06, 13.36)	146 (75, 327)
Syria	0.29 (0.11, 0.64)	3,434 (1,567, 9,000)
